# Mushrooms Adapted to Seawater: Two New Species of *Candolleomyces* (Basidiomycota, Agaricales) from China

**DOI:** 10.3390/jof9121204

**Published:** 2023-12-16

**Authors:** Kun L. Yang, Jia Y. Lin, Guang-Mei Li, Zhu L. Yang

**Affiliations:** 1 College of Forestry and Landscape Architecture, South China Agricultural University, Guangzhou 510642, China; mugoture@stu.scau.edu.cn; 2 College of Plant Protection, South China Agricultural University, Guangzhou 510642, China; mushroomflower@stu.scau.edu.cn; 3 Yunnan Key Laboratory for Fungal Diversity and Green Development, Kunming Institute of Botany, Chinese Academy of Sciences, Kunming 650201, China; liguangmei@mail.kib.ac.cn

**Keywords:** marine fungi, marine mushrooms, mangrove-associated fungi, sequestrate fungi, new taxa, phylogeny, Basidiomycota, *Candolleomyces*

## Abstract

Marine fungi have been studied for a long history in many realms, but there are few reports on marine mushrooms. In this study, marine fungi with conspicuous subglobose sequestrate basidioma were discovered from mangrove forests in South China. They grow on the deadwood of mangroves in the intertidal zone, periodically submerging into seawater due to the tide. Some marine animals were observed to nest in their basidiomata or consume them as food. The pileus-gleba-inner veil complex (PGI) of the basidioma was observed to be detached from the stipe and transferred into seawater by external forces, and drifting on sea to spread spores after maturity. The detachment mechanism of their PGIs was revealed through detailed microscopic observations. The contrast culturing experiment using freshwater and seawater potato dextrose agar media showed they have probably obligately adapted to the marine environment. Based on morphological and molecular phylogenetic evidence, two new species of *Candolleomyces* (Basidiomycota, Agaricales), namely *C. brunneovagabundus* and *C. albovagabundus*, were described. They are similar and close to each other, but can be distinguished by the size and color of the basidioma, and the size of the basidiospores.

## 1. Introduction

In a broader sense, those fungi able to grow and/or sporulate in the marine environment, or form symbiotic relationships with other marine organisms, or adapt and evolve, or be metabolically active in the marine environment are known as marine fungi [1,2]. They are widely distributed in the supralittoral, intertidal, neritic, and oceanic zones within the marine environment [3], either be saprophytic on soil, sand and bio-remains, or be symbiotic/parasitic with algae, corals, and plants, promoting the nutrient circulation and energy flow in an ecosystem [2,4,5,6]. They produce a wide variety of natural products, which are valuable resources for chemical and drug development [7]. They also kill marine wildlife and cause disease in aquaculture, bringing economic loss directly or indirectly [8,9]. Currently, marine fungi have documented 9 phyla, 33 classes, 107 orders, 273 families, 778 genera and 1947 species [10,11]. Most of them are microfungi that appear as invisible yeasts (e.g., *Yarrowia lipolytica* [12]), swimming zoospores (e.g., *Haliphthoros milfordensis* [13]), or intracellular parasites (e.g., *Glugea plecoglossi* [14]) [10,11], while the rest, although visible to naked eye, are mostly tiny ones with a diameter of no more than 2 mm, such as the marine taxa of Halosphaeriales, Pleosporales, and Xylariales [10,11,15,16,17]. Futhermore, there are many marine fungi still unknown [18,19], which have attracted increasing attention from researchers.

Of all marine fungi, marine basidiomycetes that form mushrooms are an attractive but markedly rarer group than either their terrestrial counterparts or other marine fungi, according to the current documentations [10,11]. The truly obligate members of them are mainly in two families with not yet stable boundaries in Agaricales, viz. Niaceae and Physalariaceae, including about 9 species which are relatively well studied—*Calathella mangrovei*, *Digitatispora lignicola*, *D. marina*, *Halocyphina villosa*, *Mycaureola dilseae*, *Nia epidermoidea*, *Ni. globispora*, *Ni. lenicarpa*, and *Ni. vibrissa* [20,21,22,23,24,25,26,27,28,29,30,31,32]. All of them may fruit and produce basidiospores when submerged in seawater [21,22,23,24,27,28,30], and generally share some universalities in morphology: (1) protecting the hymenium through the hydrophobic hairy surface (e.g., Ca. *mangrovei* [27], *D. lignicola* [28], *Ni. vibrissa* [21]) and/or the semi-enclosed to fully enclosed gleba (e.g., *Ha. villosa* [20], *Ni. lenicarpa* [21], *Ni. vibrissa* [21]); (2) with basidiospores more or less modified, such as appendaged (e.g., *Ni. epidermoidea* [30], *Ni. vibrissa* [21]), unusually large (e.g., *M. dilseae* [23], *D. lignicola* [28]) or wall thickened (e.g., *Ha. villosa* [20]); (3) with basidiospores generally released passively (*Ni. vibrissa* [24]; the others probably are the same but lack detailed evidence [32]). These structures may imply the evolutionary trends of marine basidiomycetes, which successfully protect their hymenium from seawater to produce spores underwater, and help the spores to be captured by substrates, or to remain active in seawater against biotic and abiotic stresses. It’s notable that Physalariaceae also includes a facultative member, viz. *Physalacria maipoensis* that produces an exposed hymenium. Inderbitzin and Desjardin (1999) [33] regarded it as a “halotolerant” species whose mycelium inoculated onto seawater media (salinity approximately 33‰) grew at the same rate as that on freshwater media, and it was not only collected in mangrove forests but also on the wood of *Lantana camara* in upland forests that are not inundated [33]. Additionally, some other lineages of basidiomycetes were also reported to contain marine fungi, such as *Fulvifomes* (Polyporales, Hymenochaetaceae), *Haloaleurodiscus* (Russulales, incertae sedis) and *Henningsomyces* (Agaricales, incertae sedis), etc., but they were mostly just found on coastal substrates without a further confirmation of whether they are obligate, facultative, or just poorly living materials [34,35,36]. There were also some basidiomycetes, reported as marine fungi, but they had just been isolated from marine environment, such as *Pleurotus pulmonarius*, *Earliella scabrosa* and *Candolleomyces candolleanus* [10,11], which were traditionally recognized as terrestrial fungi, with their real conditions in marine environment remaining to be confirmed. In summary, there are many gaps to explore regarding the diversity of marine basidiomycetes.

Mangrove forests are wetland woody plant communities in tropical and subtropical coastal intertidal zones or river estuaries [37,38], providing a unique habitat for marine basidiomycetes. In July 2023, in the Xiwan Mangrove Park and Gull Island Mangrove Park in Guangdong Province, China, we noticed that some subglobose mushrooms often appeared on the deadwood of *Sonneratia* trees there. There were also usually some stipe-like tissues around or next to these mushrooms, and the surrounding water was littered with fragments of the pileus-gleba-inner veil complex (PGI) of these mushrooms. We realized that these might be some mushrooms that, when mature, the PGI would detach from the stipe and fall into the sea to drift and spread spores, and therefore, we carried out the following studies on them: (1) investigating the detachment mechanism of the droppable PGI through detailed microscopic observations; (2) comparing their viability in freshwater and seawater by observing the spore germination rate in potato dextrose agar media (PDA) prepared with freshwater and seawater; (3) confirming their taxonomic positions through morphological examination and molecular phylogenetic analysis.

## 2. Materials and Methods

### 2.1. Specimen Collection

Specimens were collected between July 10 and October 26 in 2023, from Xiwan Mangrove Park, Shenzhen (22° 35′ N, 113° 49′ E) and Gull Island Mangrove Park, Guangzhou (22° 55′ N, 113° 32′ E) in Guangdong Province, China. Areas with a high abundance of deadwood and a low depth of water at high tide were considered as main collecting sites, covering about 6757 m^2^ in Xiwan Mangrove Park, and about 1696 m^2^ in Gull Island Mangrove Park (Figure S1). The holotypes were deposited in the Herbarium of Cryptogams, Kunming Institute of Botany, Chinese Academy of Sciences (HKAS). The isotypes, paratypes and the rest were deposited in Kun L. Yang’s private herbarium (HTBM) or completely used up. Colony samples were collected from the media (see Section 2.3) in Kun L. Yang’s laboratory and completely used up in the experiments.

### 2.2. Morphological and Ecological Observations

The macromorphological characteristics were described based on field notes and photos. Colors were confirmed and described following Yang (2023) [39]. The color card of the colors referred to can be accessed in Table S1. The micromorphological characteristics were observed on fresh and air-dried materials after sectioning and rehydrating in water or a 5% KOH solution. The notation {*a*/*b*/*c*} (*d*) *e*–*f* (*g*) [*h* ± *i*, *j*] was used to describe the size (length, width and length/width ratio (*Q*)) of the basidiospores, where the range *e*–*f* represented ≥90% of the measured values, *d* the minimum extreme value, *g* the maximum extreme value, *h* ± *i* the average value ± sample standard deviation and *j* the mode, measured from *a* basidiospores of *b* basidiomata in *c* specimens. Sections were studied using a MSD105 stereomicroscope (Murzider (Dongguan) Science and Technology Co., Ltd., Dongguan, China) and a MSD105 light microscope (Murzider (Dongguan) Science and Technology Co., Ltd., Dongguan, China) at a magnification of up to ×1000.

The photography of habitat and specimens was carried out by the built-in camera of the Xiaomi 13. To observe the potential activities of some basidiomata after they became submerged, underwater cameras (the manufacturer requested anonymity) were fixed in front of them at low tide, continuously videoing from tide rising to tide falling, and retrieved at the next low tide.

### 2.3. Contrast Culturing

To compare the viability of the two species identified in Section 2.4 and Section 2.5 in freshwater and seawater, fresh spore suspensions were made and inoculated on potato dextrose agar media (PDA) prepared with freshwater and seawater for contrast culturing. The difference of the spore germination rate in different media should reveal which environment they prefer.

#### 2.3.1. Spore Suspensions Making

One fresh, mature, and intactly preserved basidioma of the two species was collected and brought to the laboratory immediately to make fresh spore suspensions. After cutting a basidioma, the gleba fragments were picked out with a tweezer and put into a centrifuge tube with 25 mL of distilled water, oscillating for 3 min to fully release the spores into the water.

#### 2.3.2. Media Preparing

To prepare 20 freshwater PDAs, we dissolved 43 g of PDA powder (Hangzhou Microbial Reagents Co., Ltd., Hangzhou, China; containing potato infusion powder 10 g/L, dextrose 20 g/L, agar 13 g/L, chloramphenicol 0.1 g/L) in 1 L of distilled water, boiled for 2 min, and poured into 20 petri dishes. To prepare 20 seawater PDAs, we boiled 1 L of seawater from Xiwan Mangrove Park (the salinity measured 10.4‰ (≈0.18 M) by gravimetric method) for 10 min, backfilled the evaporated water, dissolved 43 g of PDA powder in it, continued boiling for 2 min, and poured into 20 Petri dishes. Before the media were solidified, we disinfected them at 60 °C for 20 min, and then cooled them to solidify. Finally, 10 freshwater PDAs and 10 seawater PDAs (both including one for control, nine for testing) were used as a group for the contrast culturing of one species.

#### 2.3.3. Culturing and Data Recording

Test groups were coated with spore suspension, while control groups were not treated. All PDAs were labeled and cultured at an environmental temperature (varying between about 25–35 °C, almost the same condition as the wild habitat of the two species). We counted the number of PDAs with colonies in each test group every 24 h. After all PDAs in test groups have emerged colonies, we stopped the culturing and observed the germinating conditions of each group. The colony samples were sequenced to verify whether they were the target species.

### 2.4. Molecular Sequencing

Genomic DNA was extracted from the voucher specimens by using the Ezup Column Fungi Genomic DNA Purification Kit (Sangon Biotech Co., Ltd., Shanghai, China). Four loci, viz. the nuclear internal transcribed spacer region (nrITS), nuclear large subunit rDNA (nrLSU), translation elongation factor 1 alpha gene (*tef-1α*), and beta tubulin gene (*β-tub*), were amplified using the primer pairs ITS1-F/ITS4 [40,41], LR0R/LR5 [42], EF1-983F/EF1-1567R [43] and B36f/B12r [44], respectively. PCR protocol and sequencing were conducted following Cai et al. (2014) [45]. The raw sequences were checked with Chromas v2.6.6 [46] and assembled with MEGA v7.0.26 [47]. The assembled sequences were deposited in GenBank with the accession numbers shown in Table S2.

### 2.5. Phylogenetic Analysis

The ITS sequences obtained from the collected specimens were blasted in the NCBI database [48] to search for similar records. In the results, the closely related records were labeled *Candolleomyces*, *Psathyrella* and *Hausknechtia*, etc., with the highest similarities around 93–94%. Therefore, we combined the nrITS, nrLSU, *tef-1α,* and *β-tub* sequences of our collected specimens, known species of *Candolleomyces* and representative species of the other 17 genera of the family Psathyrellaceae (except *Rachipsathyra,* which contains a single agaricoid species without molecular data [49]) viz. *Britzelmayria*, *Coprinellus*, *Coprinopsis*, *Cystoagaricus*, *Hausknechtia*, *Heteropsathyrella*, *Homophron*, *Iugisporipsathyra*, *Kauffmania*, *Lacrymaria*, *Narcissea*, *Olotia*, *Parasola*, *Psathyrella*, *Punjabia*, *Tulosesus,* and *Typhrasa* for molecular phylogenetic analysis (Table S2) with the Maximum Likelihood (ML) method to confirm the position of the collected specimens. DNA sequences were aligned using MAFFT v7.450 [50] and trimmed with TBtools-II v2.008 [51]. The introns of *tef-1α* and *β-tub* were removed following Wächter and Melzer (2020) [52]. The alignments of the four loci were concatenated by PhyloSuite v1.2.3 [53,54] with the missing and terminal gaps filled with “N”. The outgroups were selected following Wang et al. (2022) [55]. RaxmlGUI v2.0.10 [56] was used to find out the best-fit substitution model for the nrITS-nrLSU-*tef-1α*-*β-tub* dataset under the Akaike information criterion (AIC), and to perform an ML analysis with 1000 rapid bootstrap replicates. Nodes that received a maximum likelihood bootstrap over 50% (MLB ≥ 50%) were considered as significant supports. The phylogenetic tree was visualized with FigTree v.1.4.0 [57].

## 3. Results

### 3.1. Morphological and Ecological Observations

**Figure 1:**
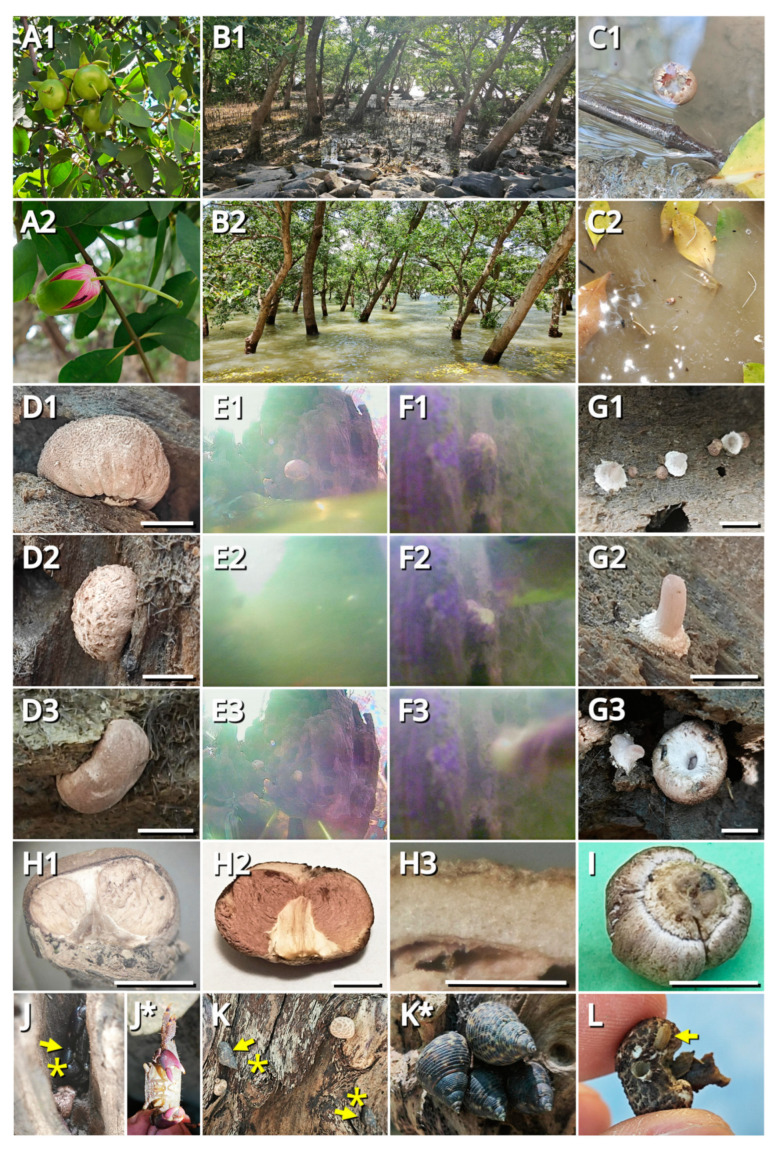
Fresh basidiomata and ecology of *Candolleomyces brunneovagabundus* (photos by Kun L. Yang). (**A1**,**A2**) The host of the holotype and paratype—*Sonneratia caseolaris* ((**A1**) Leaves and fruits; (**A2**) Leaves and a flower); (**B1**,**B2**) The main collecting site in Xiwan Mangrove Park ((**B1**) At low tide; ((**B2**) At high tide); (**C1**,**C2**) Drifting PGI occasionally seen in the collecting site ((**C1**) On a still puddle at low tide; (**C2**) On cloudy flowing seawater at high tide); (**D1**–**D3**) Basidiomata germinating from different angles of the substrate ((**D1**) From the top (HTBM1136, paratype); (**D2**) From the side (completely used up); (**D3**) From the bottom (HKAS129659, holotype); (**E1**–**E3**) Three underwater video (Video S1) captures: a wave carried away the PGI of a mature basidioma, leaving behind a stipe on the substrate; (**F1**–**F3**) Three underwater video (Video S2) captures: a fish (probably *Mugil cephalus*) came, ate an immature basidioma for 16 bites, then dragged the rest out with a fragile wood piece (substrate) and carried them away; (**G1**–**G3**) Remaining stipes on substrate ((**G1**) Naturally formed (L23180, completely used up); (**G2**) Naturally formed (L23185, completely used up); (**G3**) Manually formed by inappropriate hand-plucking when collecting the specimen (HTBM1131)); note that the stipe surface was smooth, and inner veil tissues were connected with the gleba rather than the stipe); (**H1**–**H3**) Longitudinal sections of the basidioma ((**H1**) HKAS129659 (holotype); (**H2**) HTBM1136 (paratype)); (**H3**) The spongy pileus context of HTBM1136 (paratype)); (**I**) Bottom view of a basidioma (HTBM1135); (**J**) Basidiomata (HTBM1134) associated with a crab (probably *Metopograpsus* sp., asterisked (*)); (J*) A close-up of the associated crab; (**K**) Basidiomata (HTBM1138) associated with sea snails (probably *Littoraria* sp., asterisked (*)); (K*) A close-up of the associated sea snails; (**L**) A basidioma (HTBM1218) nested a hole by a sow bug (probably *Tylos minor*). Bars: H3 = 0.5 mm, others = 5 mm.

**Figure 2:**
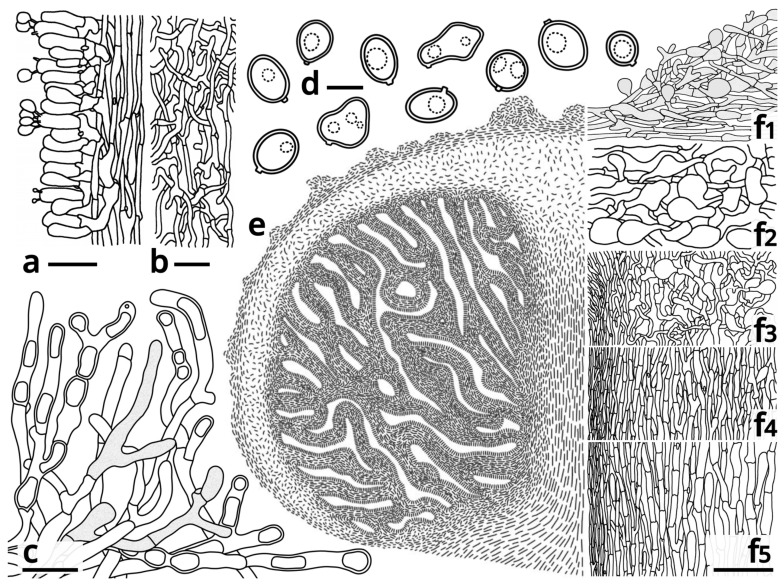
Microscopic structures of *Candolleomyces brunneovagabundus* (drawings by Kun L. Yang, from the laboratory culture L23267, others the holotype HKAS129659). (**a**) Hymenium, subhymenium and trama; (**b**) Inner veil; (**c**) Vegetative mycelium on seawater PDA; (**d**) Basidiospores; (**e**) A diagram of an immature basidioma in the longitudinal section; (**f1**–**f5**) Detailed structures of the tissues along the axis of the basidioma ((**f1**) Pileipellis and a squamule mass on pileus surface; (**f2**) Context of pileus; (**f3**) Context of pileus-stipe transition area, pellis to trama from left to right; (**f4**) Context of stipe top part, pellis to trama from left to right; (**f5**) Context of the stipe middle part, basal part almost the same as it, pellis to trama from left to right). Bars: a = 20 μm, b, c = 10 μm, d = 5 μm, f1–f5 = 50 μm.

**Figure 3:**
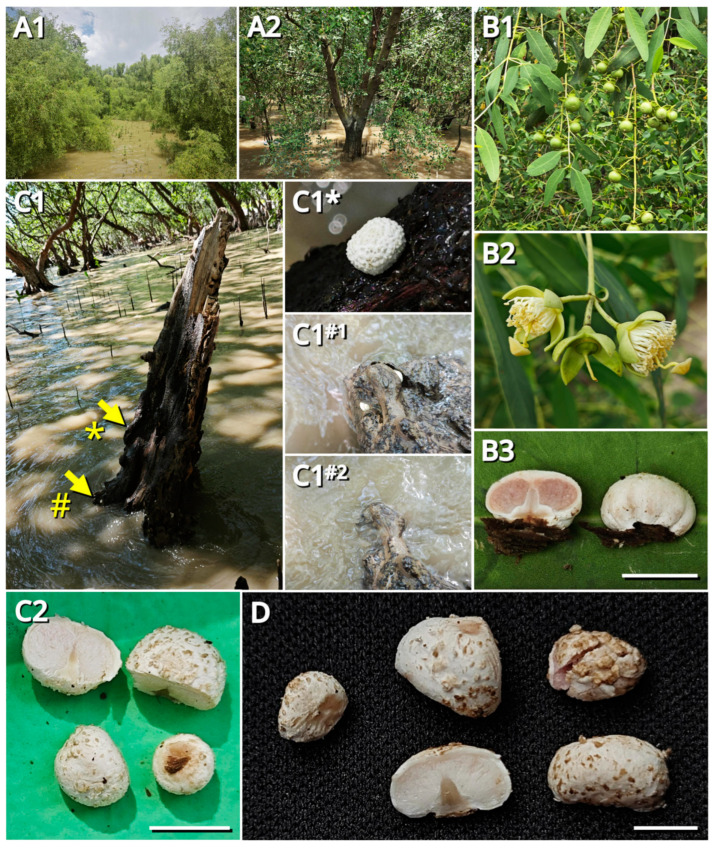
Fresh basidiomata and ecology of *Candolleomyces albovagabundus* (photos by Kun L. Yang). (**A1**,**A2**) The main collecting site in Gull Island Mangrove Park ((**A1**) Outside the forest; (**A2**) Inside the forest); (**B1**–**B3**) The holotype (HKAS129660) and its host—*Sonneratia apetala* ((**B1**) Leaves and fruits of *S. apetala*; (**B2**) Flowers of *S. apetala*; (**B3**) Basidioma of the holotype); (**C1**,**C2**) The paratype (HTBM1139) and its habitat ((**C1**) In habitat, tide rising, a non-submerged basidioma asterisked (*), three submerged basidiomata hashed (#); (C1*) A close-up of the non-submerged basidioma; (**C1^#1^**) A close-up of the basidiomata before submerged; (**C1^#2^**) A close-up of the basidiomata after submerged; (**C2**) Longitudinal sections and multi-angle views of the basidiomata; note that most of the surface brown were dirt.); (**D**) Longitudinal sections and multi-angle views of the basidiomata (HTBM1244); note that most of the brown surface was dirt. Bars: 5 mm.

**Figure 4:**
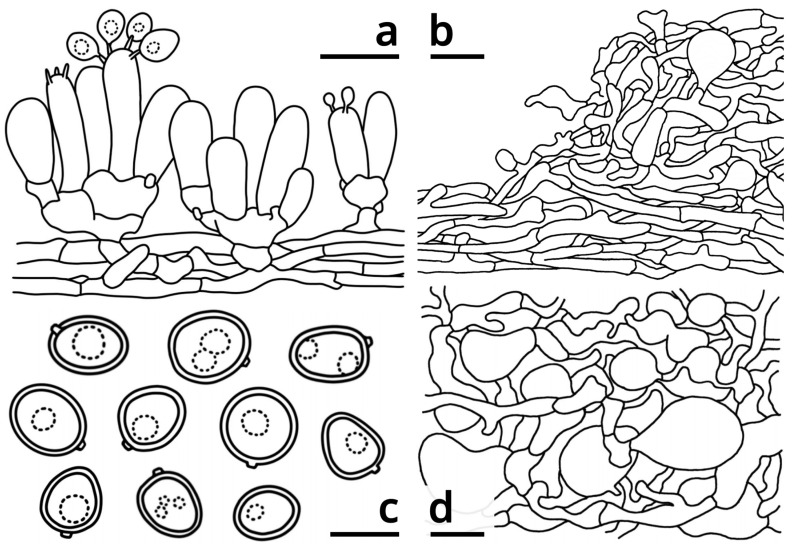
Microscopic structures of *Candolleomyces albovagabundus* (drawings by Kun L. Yang, from the holotype HKAS129660). (**a**) Hymenium and subhymenium; (**b**) Pileipellis and a squamule mass on pileus; (**c**) Basidiospores; (**d**) Context of pileus. Bars: a = 10 μm, b, d = 20 μm, c = 5 μm.

During the field study, we found that specimens representing *Candolleomyces brunneovagabundus* (see Section 3.4) were quite common in the collection site, while specimens representing *C. albovagabundus* (see Section 3.4) were less common, with the former significantly more than the latter (32 vs. 7). Figure 1 and Figure 2 show the fresh basidiomata and ecological photos, and the line drawings of the characteristics of *C*. *brunneovagabundus*. Figure 3 and Figure 4 show the fresh basidiomata and ecological photos, and the line drawings of the characteristics of *C. albovagabundus*.

#### 3.1.1. Morphological Characteristics

The collected specimens could be divided into two groups by morphology: one produced larger basidioma and a brownish pileus with brownish plasmatic pigment in the pileipellis cells, and slightly larger basidiospores, representing *Candolleomyces brunneovagabundus*; the other produced smaller basidioma and a whitish pileus with often nearly colorless cells in the pileipellis, and slightly smaller basidiospores, representing *C. albovagabundus*.

#### 3.1.2. The Droppable Pileus-Gleba-Inner Veil Complex (PGI)

For both specimens representing *Candolleomyces brunneovagabundus* and *C. albovagabundus*, stipes that lacked a pileus were common on the substrate where intact basidiomata were grown. Floating pilei with gleba and an inner veil were occasionally seen in the surrounding water, which were obviously dropped from the stipes.

It had been observed that, in these fungi, the pileus and gleba were tightly connected, the gleba and inner veil were tightly connected, and thus, the three tissues composed a continuous complex, hereinafter referred to as “pileus-gleba-inner veil complex (PGI)”, throughout the lifecycle of a basidioma. At the early development stage of a basidioma, the PGI enclosed the stipe, with its inner veil tightly attached to the stipe. However, as the basidioma matured, thet PGI slightly spread (Figure 1D1), resulting in the separation between inner veil and stipe. At this time, the only tissue connecting the PGI and the stipe was in the context of the transition area of the pileus and stipe. Detailed microscopic observations of *C. brunneovagabundus* showed that the mycelium in this area was loose and anisotropic (Figure 2e,f3), resulting in a fragile tissue that benefits the easy separation between the spread PGI and the stipe. In contrast, the mycelium that formed the stipe was compact and regularly arranged (Figure 2e,f4,f5), resulting in a tough tissue that always remains intactly on the substrate after the PGI dropped.

The common reasons of the PGI dropping were: (1) basidiomata closely beside or below the PGI becoming bigger in size as they grew up, pushing the latter upward and thus detaching from the stipe (Figure 1G1); (2) the basidioma that formed this PGI grown from the bottom of the substrate, resulting in its PGI to be dropped by gravity (Figure 1D3); (3) the basidioma that formed this PGI grown from a side of the substrate, resulting in its PGI to be carried away by waves at high tide (Figure 1E1–E3; Video S1); (4) the basidioma that formed this PGI grown from the top of the substrate, resulting in its PGI to be lifted by seawater at high tide (Figure 1G2). In addition, strong winds, heavy rain, and animal activities may also work in its dropping.

Dropped PGIs could float on seawater. It was observed that the pileus context and gleba of these fungi were spongy and filled with locules (Figure 1H1–H3 and Figure 3B3,C2,D), thus providing enough buoyancy for floating.

#### 3.1.3. Ecological Habits

For both specimens representing *Candolleomyces brunneovagabundus* and *C. albovagabundus*, the known hosts were two non-native mangrove plants introduced from the Hainan Province about 20 years ago, viz. *Sonneratia caseolaris* and *S. apetala*. These fungi grew on the remains of them, such as dead trees, stumps, driftwood, and dead aerial roots. During the sampling period of more than three months, almost all the substrates found to be infected with these fungi kept producing basidiomata in succession, round after round. It took about five days for a basidioma from the primordium developing to the PGI dropping. During this period, the basidioma was periodically submerged by seawater due to the tide, but this was not observed to hinder its growth.

There were often active animals around the basidiomata of these fungi, including many kinds of crab (e.g., *Grapsus* sp., *Metopograpsus* sp.), sea snails (e.g., *Littoraria* sp.), mudskippers (e.g., *Periophthalmus* sp.), etc. Some species, such as a kind of sow bug (probably *Tylos minor*), were found to burrow and nest in a basidioma of *C. brunneovagabundus*. During a sustained underwater observation of an immature basidioma of *C. brunneovagabundus*, a fish (probably *Mugil cephalus*) was found to consume it as food (Figure 1F1–F3; Video S2).

### 3.2. Contrast Culturing

**Figure 5:**
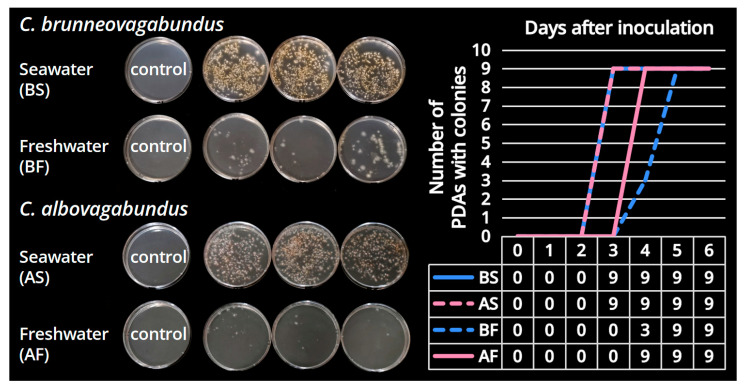
Gemination of basidiospores of *Candolleomyces brunneovagabundus* and *C. albovagabundus* on PDA cultures (each species represented by three cultures in the test group) on the sixth day after inoculation and the diagram of the number of PDAs with colonies.

Through daily observations, the diagram of the number of PDAs with colonies changed with the number of days after inoculation and the condition of PDAs in each group were shown in Figure 5. In the test groups, the colonies in seawater PDAs emerged obviously faster and denser than those in freshwater PDAs. All colonies were morphologically identical, and the sequencing results of the colony samples indicated that they were the target species. In the control groups, no colonies were observed. Such culturing results showed that the basidiospores of *Candolleomyces brunneovagabundus* and *C. albovagabundus* germinated at a significantly higher rate in seawater than in freshwater, suggesting that they are salt-preferred fungi and have probably obligately adapted to the marine environment.

### 3.3. Phylogenetic Analysis

**Figure 6:**
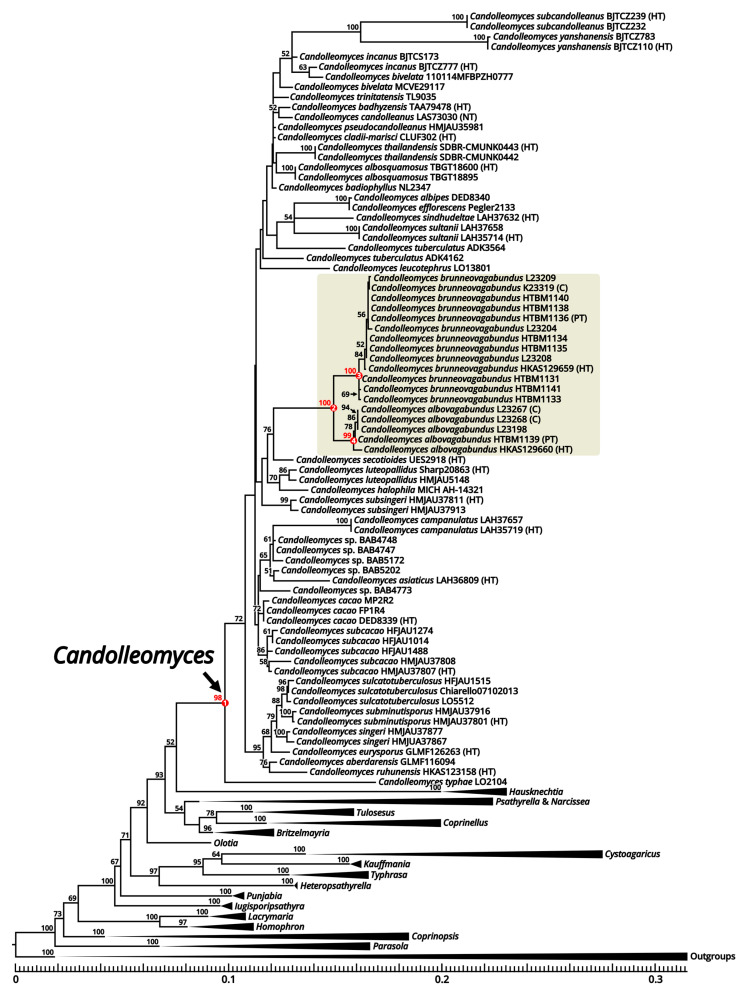
Phylogenetic tree of Psathyrellaceae inferred from concatenated nrITS-nrLSU-*tef-1α*-*β-tub* dataset. Clades of outgroups and genera were folded, except *Candolleomyces* (concerned) and *Olotia* (with only one sample). Nodes were annotated if supported by ≥50% MLB. Nodes concerned are in red and numbered. Clades of the new species are highlighted with a background color. The marks “(HT)”, “(PT)”, “(NT)” and “(C)” represent holotype, paratype, neotype and colony sample, respectively.

A total of 215 nrITS sequences, 180 nrLSU sequences, 125 *tef-1α* sequences, and 122 *β-tub* sequences from specimens and colony samples, including 18 nrITS sequences, 17 nrLSU sequences, 8 *tef-1α* sequences, and 3 *β-tub* sequences newly generated from 18 specimens that we collected were used in the phylogenetic analysis, employing the GTR+G+I model as the best-fit substitution model. The concatenated nrITS-nrLSU-*tef-1α*-*β-tub* dataset contained 3341 nucleotides in length, including 719 of nrITS, 1341 of nrLSU, 913 of *tef-1α,* and 368 of *β-tub* (Alignment S1). The phylogenetic tree generated from ML analysis was displayed in Figure 6 with the clades of outgroups and genera except *Candolleomyces* (concerned) and *Olotia* (with only one sample) folded. For the original tree with all unfolded clades, see Figure S6.

In the current phylogeny (Figure 6), our specimens stably nested in the genus *Candolleomyces* supported by 98% MLB (node 1 in Figure 6) and grouped together as a distinct independent lineage from other sampled *Candolleomyces* species supported by 100% MLB (node 2 in Figure 6). Inside this clade, two subclades clearly separated with a genetic distance of about 1.92% between them, one supported by 100% MLB (node 3 in Figure 6) and the other by 99% MLB (node 4 in Figure 6), suggesting that our specimens represent two new species of *Candolleomyces*, and they are sister to each other. One colony of *C. brunneovagabundus* and two colonies of *C. albovagabundus* clustered with the collections of the corresponding species, respectively (Figure 6), indicating that there were no contaminants in the spore suspensions.

### 3.4. Taxonomy

*Candolleomyces brunneovagabundus* Kun L. Yang, Jia Y. Lin & Zhu L. Yang, sp. nov. (Figure 1, Figure 2, Figure S2 and Figure S3)

Registration identifier: FN571695

Etymology: *brunneo-*, brown, in reference to the brownish basidioma of this fungus; *vagabundus*, wandering, in reference to the pileus-gleba-inner veil complex of this fungus drifting on sea after release from the stipe.

Diagnosis: Differs from the other known *Candolleomyces* species in its brownish sequestrate basidoma and marine habits.

Type: China, Guangdong Province, Shenzhen, Bao’an District, 22° 35′ 57.87″ N, 113° 49′ 34.68″ E, on deadwood of *Sonneratia caseolaris* in mangrove forest, elevation 0 m, July 10, 2023, Kun L. Yang & Jia Y. Lin, L23178 (HKAS129659, holotype! (nrITS: OR711031; nrLSU: OR711047; *tef-1α*: OR791600); HTBM1130, isotype!); same location, 12 July 2023, Kun L. Yang & Jia Y. Lin, L23184 (HTBM1136, paratype! (nrITS: OR711036; nrLSU: OR711052)).

Description: Basidioma tiny to small, sequestrate, mostly growing from the top or sides of the substrate, rarely from the bottom of the substrate, oblately subglobose, 7.5–17 mm in diameter, 6–10 mm in height when mature. Pileus enclosing the other parts of the basidioma, often with wrinkles on the margin, tightly attaching to the stipe base when young, slightly spreading, separating from the stipe base and easily detaching from the stipe top when mature, peach white (#FAF1EC) to wafer red (#E4D4CD), covered with silk brown (#CAB7AA) to beaver brown (#9D7B69), sometimes with a mulberry pink (#CE5086) tinge, granular, warty to felted squamules. Context of the pileus more or less spongy, with tiny locules, ceramic white (#FEFEFA), merino white (#F9F5EC) to medium gray (#E0DEDD), without color change when injured. Gleba sublamellate to spongy, with labyrinthine locules, lotus-root orange (#F5E9D9) to bone brown (#E3D3C4) when young, thatch red (#BEA39C) to sandal red (#BA8A73) when mature. Stipe ceramic white (#FEFEFA), merino white (#F9F5EC) to medium gray (#E0DEDD), sometimes with a wafer red (#E4D4CD) tinge, becoming darker after being soaked in seawater, upper part coniform to nearly cylindrical, base suction-cup-shaped. Context of the stipe with longitudinal texture, concolorous with the context of the pileus, becoming darker after being soaked in seawater. Inner veil (the interlayer between gleba and stipe) pure white (#FFFFFF) to ceramic white (#FEFEFA), very thin, tightly connecting with the gleba, attaching the stipe when immature, separating from the stipe as the pileus with the gleba slightly spreading when mature. Odor indistinct. Taste mild.

Basidia clavate to subcylindrical, 2-spored or 3-spored, rarely 4-spored, slightly thick-walled, nearly colorless in both water and KOH, 12–21.5 × 5–6 μm, surrounding by basidioles measured 12–24.5 × 5–6.5 μm. Basidiospores very variable in shape, generally subglobose to ellipsoid, sometimes globose, oblong, ovoid, obovoid, lacrymoid, amygdaliform, fusiform, phaseoliform, triangular, heart-shaped, or ellipsoid with a median constriction, etc., slightly thick-walled, smooth, more or less tinged licorice brown (#BFB075) in both water and KOH, {40/4/3} 6–9 (10) [7.29 ± 1.04, 6.00] × (4.5) 5–6.5 (7) [5.82 ± 0.59, 6.00] μm, Q = (1.00) 1.06–1.58 (2.00) [1.26 ± 0.21, 1.55], inamyloid, with a distinct apiculus, without a germ pore. Pleurocystidia absent. Pileipellis a cutis to intricate trichoderm, composed of usually slightly thick-walled, sometimes thick-walled, tinged licorice brown (#BFB075) in both water and KOH, compactly and regularly arranged to interwoven, moderately to frequently branching hyphae with abundant clamps and moderate to abundant inflated cells measured 15–35.5 × 10.5–17 μm. Tramal plate 50–90 μm thick, composed of 2–7 μm wide, thin-walled, nearly colorless in both water and KOH, compactly and regularly arranged, moderately branching hyphae with abundant clamps and scarce inflated terminals. Context of the pileus spongy, composed of 2–8.5 μm wide, thin- to thick-walled, colorless in both water and KOH, loose, interwoven, frequently branching hyphae with abundant clamps and very abundant inflated cells measured 11.5–48.5 × 8–27 μm. Context of the pileus-stipe transition area composed of 2.5–8.5 μm wide, thin-walled, colorless in both water and KOH, loose, interwoven, frequently branching hyphae with abundant clamps and abundant inflated terminals. Context of the stipe top part composed of 2–8.5 μm wide (increasing from pellis to trama), thin-walled, colorless in both water and KOH, somewhat loose, regularly arranged to interwoven, moderately to frequently branching hyphae with scarce clamps and moderate inflated terminals. Context of the stipe middle part composed of 3–9 μm wide (increasing from pellis to trama), thin-walled, colorless in both water and KOH, compactly and regularly arranged, moderately branching hyphae with scarce inflated terminals and clamps abundant in pellis, scarce in trama. Context of the stipe basal part composed of 3.5–9 μm wide (increasing from pellis to trama), thin-walled, colorless in both water and KOH, compactly and regularly arranged, moderately branching hyphae with scarce clamps and scarce inflated terminals. Inner veil composed of 2–3 μm wide, thin-walled, colorless in both water and KOH, loose, interwoven, frequently branching hyphae with very abundant clamps and scarce inflated terminals.

Mycelium on seawater PDA pure white (#FFFFFF) at first, becoming more or less ginger yellow (#ECD86C) to loquat orange (#F3D390) after 3–5 days, with a deeper back color; 4-day old primary mycelium and secondary mycelium almost identical in thickness range, 2–6 μm wide, frequently branching and curving, with an undulating wall and abundant large vacuoles.

Habitat: Growing in groups on deadwood in mangrove forests dominated by trees of *Sonneratia caseolaris* and *S. apetala*. Currently known from South China (Guangdong Province, Shenzhen).

Additional specimens examined: China, Guangdong Province, Shenzhen, Bao’an District, 22° 35′ 57.87″ N, 113° 49′ 34.68″ E, on deadwood of *Sonneratia caseolaris* in mangrove forest, elevation 0 m, July 12, 2023, Kun L. Yang & Jia Y. Lin, L23179 (HTBM1131 (nrITS: OR711032; nrLSU: OR711048)), L23180 (completely used up), L23181 (HTBM1133 (nrITS: OR711033; nrLSU: OR711049)), L23182 (HTBM1134 (nrITS: OR711034; nrLSU: OR711050)), L23183 (HTBM1135 (nrITS: OR711035; nrLSU: OR711051)), L23185 (completely used up), L23186 (HTBM1138 (nrITS: OR711037; nrLSU: OR711053)), L23188 (HTBM1140 (nrITS: OR711039; nrLSU: OR711055)), L23189 (HTBM1141 (nrITS: OR711040; nrLSU: OR711056)); same location, July 16, 2023, Kun L. Yang & Jia Y. Lin, L23192 (completely used up); same location, July 20, 2023, Kun L. Yang & Jia Y. Lin, L23197 (HTBM1149), L23199 (completely used up), L23202–L23203 (completely used up), L23204 (completely used up (nrITS: OR711043; nrLSU: OR711059; *tef-1α*: OR727287; *β-tub*: OR727290)), L23205–L23207 (completely used up), L23208 (completely used up (nrITS: OR711044; nrLSU: OR711060; *tef-1α*: OR727288; *β-tub*: OR727291)), L23208 (completely used up (nrITS: OR711045 nrLSU: OR711061; *tef-1α*: OR727289; *β-tub*: OR727292)); same location, Aug. 6, 2023, Kun L. Yang & Jia Y. Lin, L23260 (HTBM1212), L23261 (HTBM1213), L23262 (HTBM1214), L23264 (HTBM1216), L23265 (HTBM1217), L23266 (HTBM1218); same location but on deadwood of *Sonneratia apetala* in mangrove forest, Sep. 16, 2023, Kun L. Yang & Jia Y. Lin, K23371 (HTBM1243); same location, Sep. 17, 2023, Kun L. Yang & Jia Y. Lin, K23374 (HTBM1246); same location, Sep. 24, 2023, Kun L. Yang & Jia Y. Lin, K23376 (completely used up); same location, Oct. 26, 2023, Kun L. Yang & Jia Y. Lin, L23486 (HTBM1467). Cultures in Kun L. Yang’s laboratory (completely used up): July 26, 2023, K23319 (nrITS: OR791048).

Comments: *Candolleomyces brunneovagabundus* is similar and close to *C. albovagabundus*, but differs from the latter by brownish, larger basidioma, and slightly larger basidiospores. The nrITS sequence difference between them is about 2.6–3.2%.

*Candolleomyces albovagabundus* Kun L. Yang, Jia Y. Lin & Zhu L. Yang, sp. nov. (Figure 3, Figure 4, Figure S4 and Figure S5)

Registration identifier: FN571696

Etymology: *albo-*, white, in reference to the whitish basidioma of this fungus; *vagabundus*, wandering, in reference to the pileus-gleba-inner veil complex of this fungus drifting on sea after released from the stipe.

Diagnosis: Differs from other known *Candolleomyces* species in its whitish sequestrate basidoma and marine habit.

Type: China, Guangdong Province, Guangzhou, Panyu District, 22° 55′ 03.06″ N, 113° 32′ 44.30″ E, on deadwood of *Sonneratia apetala* in mangrove forest, elevation 0 m, July 16, 2023, Kun L. Yang & Jia Y. Lin, L23191 (HKAS129660, holotype! (nrITS: OR711041; nrLSU: OR711057; *tef-1α*: OR727285); HTBM1143, isotype!); China, Guangdong Province, Shenzhen, Bao’an District, 22° 35′ 57.87″ N, 113° 49′ 34.68″ E, on deadwood of *Sonneratia caseolaris* in mangrove forest, elevation 0 m, July 12, 2023, Kun L. Yang & Jia Y. Lin, L23187 (HTBM1139, paratype! (nrITS: OR711038; nrLSU: OR711054)).

Description: Basidioma tiny to small, sequestrate, growing from the top, sides or bottom of the substrate, oblately subglobose, 7.5–9 mm in diameter, 5–5.5 mm in height when mature. Pileus enclosing the other parts of basidioma, often with wrinkles on margin, tightly attaching to the stipe base when young, slightly spreading, separating from the stipe base and easily detaching from the stipe top when mature, pure white (#FFFFFF) to ceramic white (#FEFEFA), covered with concolorous to palely licorice brown (#BFB075), warted to lumpy squamules. Context of the pileus more or less spongy, with tiny locules, ceramic white (#FEFEFA), merino white (#F9F5EC) to medium gray (#E0DEDD), without a color change when injured. Gleba sublamellate to spongy, with labyrinthine locules, ceramic white (#FEFEFA) to lotus-root orange (#F5E9D9) when young, bone brown (#E3D3C4) to thatch red (#BEA39C) when mature. Stipe ceramic white (#FEFEFA), merino white (#F9F5EC) to mist brown (#DCD8C9), upper part coniform to nearly cylindrical, base suction-cup-shaped. Context of the stipe with longitudinal texture, concolorous with the context of pileus, becoming darker after soaked in seawater. Inner veil (the interlayer between gleba and stipe) pure white (#FFFFFF) to ceramic white (#FEFEFA), very thin, tightly connecting with the gleba, attaching the stipe when immature, separating from the stipe as the pileus with gleba slightly spreading when mature. Odor indistinct. Taste mild.

Basidia clavate to subcylindrical, 2-spored or 3-spored, rarely 4-spored, slightly thick-walled, nearly colorless in both water and KOH, 12–19 × 4.5–7 μm, surrounding by basidioles measured 11.5–19 × 5–6.5 μm. Basidiospores variable in shape, generally subglobose to ellipsoid, sometimes globose, oblong, ovoid, obovoid, rectangular, amygdaliform, fusiform, heart-shaped or ellipsoid with median constriction, etc., slightly thick-walled, smooth, more or less tinged licorice brown (#BFB075) in both water and KOH, {40/3/2} (5) 5.5–8 [6.52 ± 0.71, 6.50] × 4.5–6 (6.5) [5.40 ± 0.52, 5.50] μm, Q = (1.00) 1.02–1.60 (1.78) [1.23 ± 0.19, 1.30], inamyloid, with a distinct apiculus, without a germ pore. Pleurocystidia absent. Pileipellis a cutis to intricate trichoderm, composed of thin-walled to slightly thick-walled, sometimes thick-walled, nearly colorless to palely tinged licorice brown (#BFB075) in both water and KOH, compactly and regularly arranged to interwoven, moderately to frequently branching hyphae with abundant clamps and scarce to moderate inflated cells measured 13.5–40 × 9–35.5 μm. Tramal plate 55–85 μm thick, composed of 2.5–6 μm wide, thin-walled, nearly colorless in both water and KOH, compactly and regularly arranged, moderately branching hyphae with abundant clamps and scarce inflated terminals. Context of the pileus spongy, composed of 1–7.5 μm wide, thin- to thick-walled, colorless in both water and KOH, loose, interwoven, frequently branching hyphae with scarce clamps and very abundant inflated cells measured 17–32 × 9–28.5 μm.

Mycelium on seawater PDA pure white (#FFFFFF) at first, becoming more or less ginger yellow (#ECD86C) to loquat orange (#F3D390) after 4–6 days, with a deeper back color; 5-day old primary mycelium and secondary mycelium almost identical in thickness range, 2–6 μm wide, frequently branching and curving, with an undulating wall and abundant large vacuoles.

Habitat: Growing in groups on deadwood in mangrove forests dominated by trees of *Sonneratia caseolaris* and *S. apetala*. Currently known from South China (Guangdong Province, Guangzhou and Shenzhen).

Additional specimens examined: China, Guangdong Province, Guangzhou, Panyu District, 22° 55′ 03.06″ N, 113° 32′ 44.30″ E, on deadwood of *Sonneratia apetala* in mangrove forest, elevation 0 m, July 16, 2023, Kun L. Yang & Jia Y. Lin, L23192 (completely used up); China, Guangdong Province, Shenzhen, Bao’an District, 22° 35′ 57.87″ N, 113° 49′ 34.68″ E, on deadwood of *Sonneratia caseolaris* in mangrove forest, elevation 0 m, July 20, 2023, Kun L. Yang & Jia Y. Lin, L23198 (completely used up (nrITS: OR711042; nrLSU: OR711058; *tef-1α*: OR727286)); same location, 6 August 2023, Kun L. Yang & Jia Y. Lin, L23263 (HTBM1215); same location, Sep. 17, 2023, Kun L. Yang & Jia Y. Lin, K23372 (HTBM1244), K23375 (HTBM1247); same location, 24 September 2023, Kun L. Yang & Jia Y. Lin, K23377 (completely used up). Cultures in Kun L. Yang’s laboratory (completely used up): 11 August 2023, L23267 (nrITS: OR782703; nrLSU: OR782811; *tef-1α*: OR791601); 12 August 2023, L23268 (nrITS: OR782704; nrLSU: OR782812; *tef-1α*: OR791602), L23269–L23270; 14 August 2023, L23272–L23283.

Comments: *Candolleomyces albovagabundus* is similar and close to *C. brunneovagabundus*, but differs from the latter by whitish, smaller basidioma and slightly smaller basidiospores. The nrITS sequence difference between them is about 2.6–3.2%.

## 4. Discussion

### 4.1. Two New Species of Candolleomyces

*Candolleomyces* is a common genus of small- to medium-sized saprophytic agarics, split from *Psathyrella* s. l. by Wächter and Melzer (2020) [52] with the guide of molecular phylogeny. In such a genus, *C. brunneovagabundus* and *C. albovagabundus* that produce sequestrate basidioma seem to be unique, but are not the first reported. *Candolleomyces secotioides* reported from Sonoran desert, Mexico used to be the only sequestrate species in this genus [58], now clustering with *C. brunneovagabundus* and *C. albovagabundus* in the phylogeny although with only 76% MLB (Figure 6). All the three species produce a semi-closed to fully closed basidioma that is good at maintaining moisture, but probably have different origins. The sequestrate basidioma of *C. secotioides* may be an adaptation to environmental drought, stressed by water scarcity due to the poor precipitation and high evaporation, while those of *C. brunneovagabundus* and *C. albovagabundus* may be adaptations to physiological drought, stressed by the difficulty of absorbing water in high salinity environment. These two different ways of drought created two different evolutionary fates, may be the cause of the differentiation of *C. secotioides*, *C. brunneovagabundus* and *C. albovagabundus*. Additionally, *C. halophilus* reported from salt-marshy areas in Mallorca, Spain is another haloduric species in this genus, but it produces typical agaricoid basidioma [59,60]. It has also not escaped our notice that *C. candolleanus* was reported to be isolated from the marine sponge *Dragmacidon reticulatum* and the zoanthid *Palythoa haddoni* in Calabon et al. (2023) [10,11], but whether this terrestrially common fungus is metabolically active in marine environment, or it is just a contaminant from soil materials in terrestrial runoff absorbed by marine organisms remains unknown. In the genus *Coprinopsis*, *Cop. pulchricaerulea* reported from Australia also produce sequestrate basidioma similar to *C. brunneovagabundus* and *C. albovagabundus* in structure [61]. However, the inner veil of them is different, as the one of *Cop. pulchricaerulea* tightly connects with the stipe rather than the gleba as seen in the published figures [61], while that of *C. brunneovagabundus* and *C. albovagabundus* tightly connects with the gleba rather than the stipe (Figure 1G3).

*Candolleomyces brunneovagabundus* and *C. albovagabundus* create a droppable dissemination unit by producing anisotropic mycelium in the transition area of stipe and pileus. This phenomenon looks special, but is also found in some other agarics, such as the stilboid fruit body of *Mycena citricolor*—a pathogenic fungus causing the disease commonly known as American Leaf Spot on coffee plants [62]. However, the stilboid fruit body of this fungus may be an adaptation to the host as its dropped pileus will roll down with gravity, which may help it attack the lower plant tissues and increase the ability to infect plants.

### 4.2. A New Lineage of Marine Basidiomycetes

**Figure 7:**
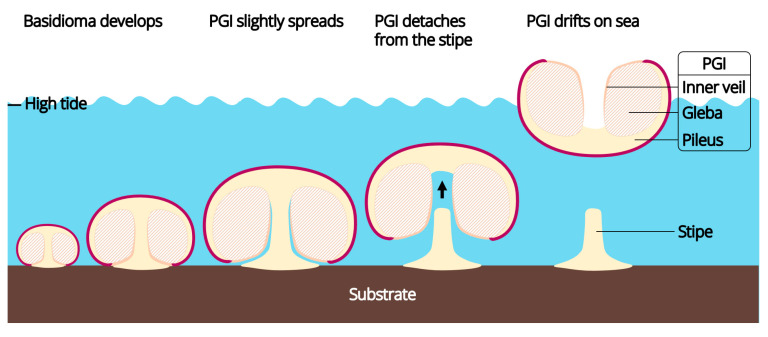
Development of the dissemination unit—pileus-gleba-inner veil complex (PGI) of *Candolleomyces brunneovagabundus* and *C. albovagabundus*.

*Candolleomyces brunneovagabundus* and *C. albovagabundus* produce basidioma with a conspicuous size, salt-preferred habit, droppable and floatable PGIs, implying that they are marine mushrooms that have probably obligately adapted to marine environment, adding a new lineage from Psathyrellaceae to marine basidiomycetes. They are able to fruit and produce basidiospores when submerged in seawater and the contrast culturing showed that they are probably obligate to the marine environment, which are similar to the marine members of Niaceae and Physalariaceae. *C*. *brunneovagabundus* and *C. albovagabundus* adopt a similar morphology with them, in which their basidioma abandons the agaricoid plesiomorphy of an exposed hymenium, incubating the hymenium in a seawater-isolated cavity enclosed by pileus and inner veil, thus adapting to fruit and producing basidiospores in a submerged environment (Figure 7). They also form droppable and floatable dissemination units that further facilitates their dispersal in seawater (Figure 7), and the similar conditions were also observed or predicted in the gastroid species such as *Nia* spp. in seawater and *Limnoperdon incarnatum* in freshwater [27,63]. Moreover, the dissemination units dropped by these fungi may not just about drifting on water, because they are likely to be eaten by aquatic animals, along with the activities of them to spread more quickly and widely, and thus into new habitats. One of our field observations noticed a fish eating the basidioma of *C. brunneovagabundus* (Figure 1F1–F3; Video S2). The fish was morphologically identified as *Mugil cephalus*, a kind of omnivorous fish that widely distributes in tropical to temperate waters [64]. These blackish fish are common and abundant in the coastal areas of Shenzhen and Guangzhou, sometimes making the coastal waters looked blackish when gathering for food. Given the speed and range of the activities of these fish, they may be one of the powerful disseminators of these fungi. Furthermore, the basidiospores of *C. brunneovagabundus* and *C. albovagabundus* also have a thicker wall compared with their terrestrial counterparts, which may help them stay active longer in seawater or the digestive tract of animals. Through these innovations, *C. brunneovagabundus* and *C. albovagabundus* completed the transition from terrestrial to aquatic environments, invading marine habitats.

Jones and Choeyklin (2008) [32] mentioned that, “typically large putrescent fruit bodies of aquatic basidiomycetes are ‘impractical’”. This is evident for the known obligate marine basidiomycetes as they commonly produce reduced basidioma which rarely exceed 2 mm in size [20,21,22,23,24,25,26,27,28,29,30,31,32]. Although the basidioma of *Candolleomyces brunneovagabundus* with a pileus diameter of up to 1.7 cm set a new record, it’s still a small size comparing with the remarkable basidiomycetes on land [65]. However, in freshwater basidiomycetes, *Psathyrella aquatica* reported from North America produces basidioma up to 10 cm in height underwater in flowing stream [66]. It has developed lamellae with an exposed hymenium, resulting in its basidiospores released into the gas pocket under pileus and carried away by water or aquatic animals, successfully adapting to the submerged environment in an agaricoid form. It implies that the forms of basidiomycetes adapted to aquatic environments may be diverse.

### 4.3. Are They Native to the Collection Sites?

*Candolleomyces brunneovagabundus* and *C. albovagabundus* are currently only found on the remains of *Sonneratia* spp., and the contrast culturing in freshwater and seawater showed they are salt-preferred fungi, suggesting their distribution areas may be limited to mangrove forests, even those containing *Sonneratia* species. However, only the Hainan Province in China has a natural distribution of *Sonneratia* plants, and those in other areas were introduced from Hainan in recent decades through saplings or seeds, including the two mangrove forests in Guangdong Province investigated in this study [67,68,69,70]. Therefore, it is speculated that the *C. brunneovagabundus* and *C. albovagabundus* found in Guangdong Province may be: (1) native species in the *Sonneratia* forests in Hainan, but carried to Guangdong by the *Sonneratia* saplings introduced from Hainan, or by the activities of marine animals; (2) native species in the native mangrove forests in Guangdong dominated by *Acanthus ilicifolius*, *Aegiceras corniculatum*, *Avicennia marina* and *Kandelia obovata*, etc., but after the introduction of *Sonneratia* plants, still adapted and turned them into new hosts. Although we carefully examined a variety of other mangrove plants in the collection sites and neither of the two fungi was found, a more extensive sampling in the future can further confirm whether they are obligate to *Sonneratia* spp., and to discover new distribution areas of them.

### 4.4. Further Testing of the Salt Tolerance

In the contrast culturing experiment in this study, we only confirmed that *Candolleomyces brunneovagabundus* and *C. albovagabundus* prefer seawater with a salinity of 10.4‰ (≈0.18 M) from the habitat instead of freshwater. This raises some questions that could be further studied in the future, such as (1) What is the optimal salinity for their growth? (2) What is the highest salinity that they can tolerate? (3) Is their viability affected by other environmental factors such as pH and temperature?, etc. Additionally, considering that some researchers have specifically defined the words “halophilic” and “halotolerant” in mycological sense, for example, Gunde-Cimerman et al. (2005) [71,72] defined that those fungi that can grow in vitro at 3 M salt concentrations and that are regularly isolated from global environments at salinities above 1.7 M are characterized as “halophilic”, whereas the sporadic isolates that can grow in vitro at 3 M NaCl are considered as “halotolerant”, making these two terms seem more applicable to fungi in relatively “extreme” high-salt environments. Until the optimal growth salinity and extreme growth salinity of *C. brunneovagabundus* and *C. albovagabundus* are further determined, we suggest to avoid using the terms “halophilic” or “halotolerant” to describe them.

## Data Availability

The sequences generated in this study are available in NCBI GenBank (https://www.ncbi.nlm.nih.gov/genbank, accessed on 1 November 2023) under the accession numbers shown in Table S1. The specimens studied in this study were deposited in the Herbarium of Cryptogams, Kunming Institute of Botany, Chinese Academy of Sciences (HKAS) and Kun L. Yang’s private herbarium. Supplementary Materials were deposited in Zenodo (https://zenodo.org/, accessed on 1 November 2023), see section Supplementary Materials for details.

## Supplementary Materials

Supplementary materials in this study were deposited in Zenodo (https://doi.org/10.5281/zenodo.10060712), including the following items: Alignment S1. Alignment of the phylogenetic tree in this study, containing 3341 nucleotides in length, including 719 of nrITS, 1341 of nrLSU, 913 of *tef-1α* and 368 of *β-tub* arranged in order of nrITS-nrLSU-*tef-1α*-*β-tub*, representing 216 specimens (205 of ingroups and 11 of outgroups); Figure S1. Main collecting sites in Xiwan Mangrove Park and Gull Island Mangrove Park (Map sources: National Platform for Common Geospatial Information Services [73]); Figure S2. Basidiopsores of *Candolleomyces brunneovagabundus* (HTBM1218) under light microscope at a magnification of × 1000; Figure S3. Pileipellis elements of *Candolleomyces brunneovagabundus* (HTBM1130 (isotype)) under light microscope at a magnification of ×400; Figure S4. Basidiopsores of *Candolleomyces albovagabundus* (HTBM1143 (isotype)) under light microscope at a magnification of ×1000; Figure S5. Pileipellis elements of *Candolleomyces albovagabundus* (HTBM1139 (paratype)) under light microscope at a magnification of ×400; Figure S6. The original unfolded version of the tree in Figure 6; Table S1. Color card of the colors referred in this study. The color names were designated following the color nomenclatural code for description works of specimens in Kun L. Yang’s private herbarium (HTBM) (Yang 2023 [39]); Table S2. Information of the specimens used in phylogenetic analysis. The specimens sequenced in this study are in bold. The marks “(HT)”, “(IT)”, “(PT)”, “(ET)” and “(NT)” represent holotype, isotype, paratype, epitype and neotype, respectively; Video S1. A wave carried away the PGI of a mature basidioma of *Candolleomyces brunneovagabundus*, leaving behind a stipe on the substrate; Video S2. A fish (probably *Mugil cephalus*) ate an immature basidioma of *Candolleomyces brunneovagabundus* for 16 bites, then dragged the rest out together with a fragile wood piece (substrate) and carried them away. References [74,75,76,77,78,79,80,81,82] are cited in the supplementary materials.
